# Supplementary material for: Abrupt drainage basin reorganization following a Pleistocene river capture
Source: Nat Commun. 2018 Sep 14;9:3756. doi: 10.1038/s41467-018-06238-6 (PMC6138651; doi:10.1038/s41467-018-06238-6)
Supplement: Supplementary file 1 — Supplementary Information [file 41467_2018_6238_MOESM1_ESM.pdf]

SUPPLEMENTARY

Abrupt drainage basin reorganization following a Pleistocene river capture

Fan et al.

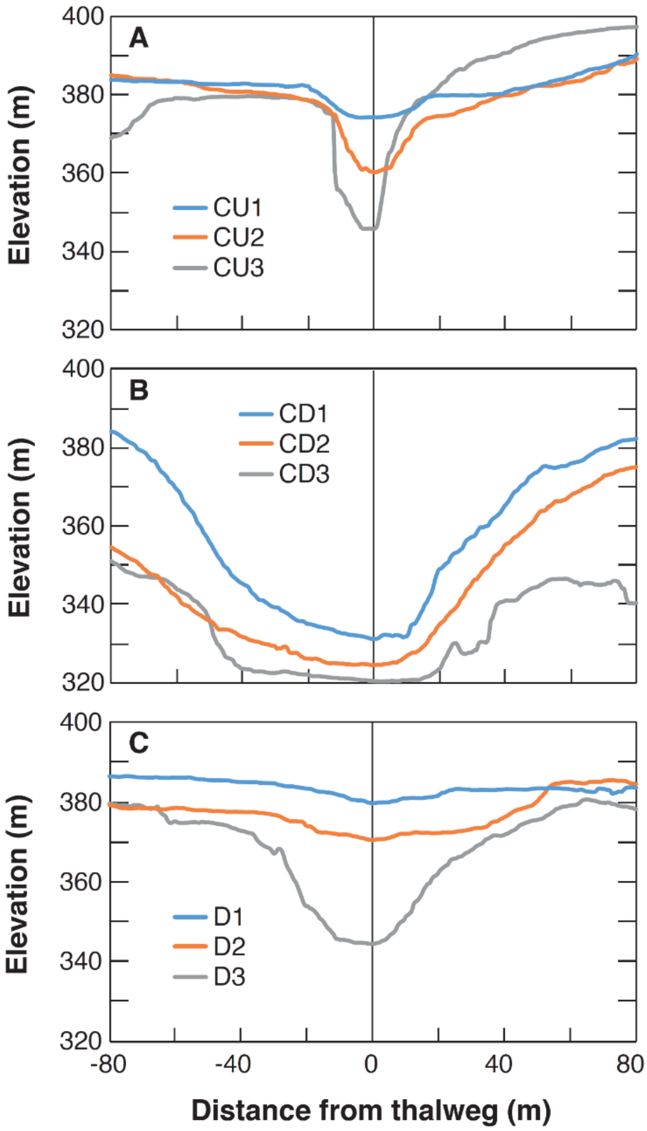

Supplementary Figure 1 | Cross-sectional profiles for both the Chaiwen and Daotang Rivers from our UAV-based high-resolution (0.5 m) DEM. a, Cross-sectional profiles of the Chaiwen River upstream of the elbow. **b,** Cross-sectional profiles of the Chaiwen River downstream of the elbow. **c,** Cross-sectional profiles of the Daotang River. The abbreviations CU, CD and D refer to cross-sectional profiles located on the upstream portion of the Chaiwen River, the downstream portion of the Chaiwen River and the Daotang River, respectively.

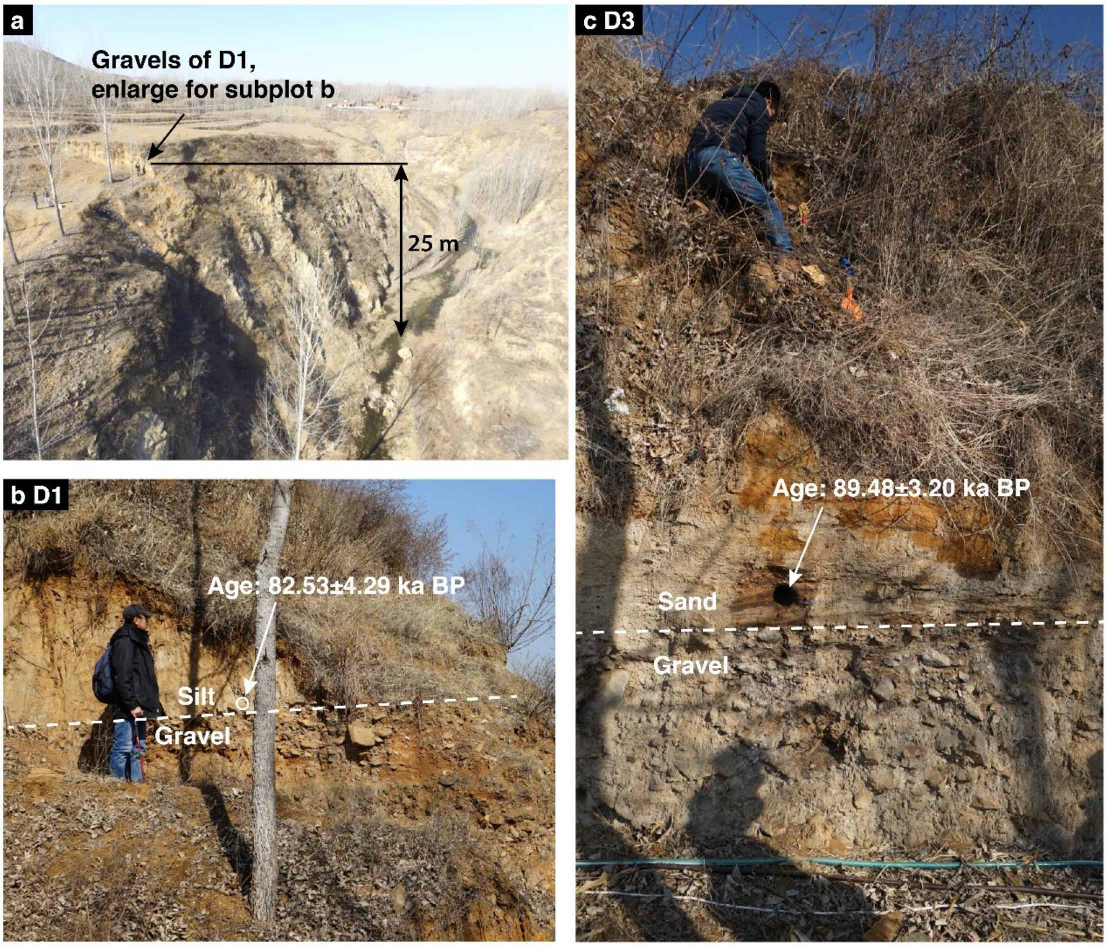

Supplementary Figure 2 | Photographs showing the vertical sections in D1 and D3. a, Overview of D1, which is 25 m higher than the present Daotang riverbed. **b,** Detailed photograph of vertical section in D1; the bed of imbricated cobbles was covered by a silt deposit, and the silt was dated to be 82.53±4.29 ka BP. **c,** Detailed photograph of vertical section in D3; the bed of imbricated cobbles was covered by a sand deposit, and the sand was dated to be 89.48±3.03 ka BP.

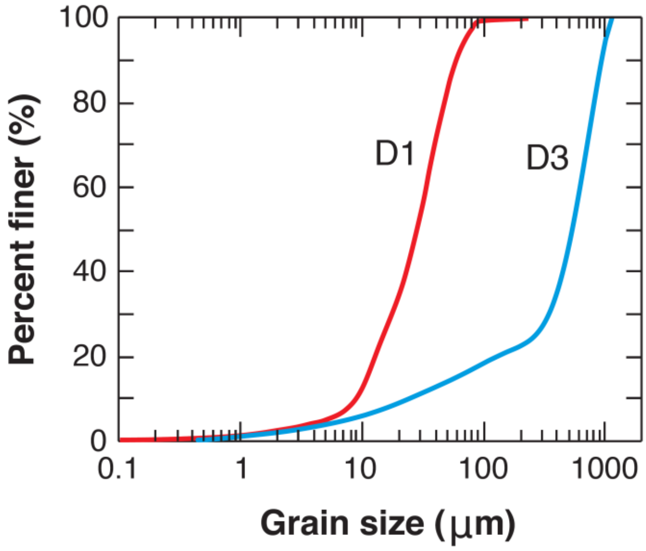

Supplementary Figure 3 | Grain size distribution of the dated material in D1 and D3.

Supplementary Table 1 | Information of the samples for OSL dating

Sample	Depth (m)	Water Content (%)	U(ppm)	Th(ppm)	K(%)	Dose rate (Gy/ka)	Equal Dose (Gy)	Age (ka BP)
D1	1.5	6 ±3	3.61 ±0.15	30.32 ±0.60	2.18 ±0.02	6.56 ±0.27	541.73 ±17.01	82.53 ±4.29
D3	3.0	5 ±2	2.2 ±0.09	8.20 ±0.16	3.75 ±0.04	5.04 ±0.16	451.24 ±4.60	89.48 ±3.03
