# Supplementary material for: Abrupt drainage basin reorganization following a Pleistocene river capture
Source: Nat Commun. 2018 Sep 14;9:3756. doi: 10.1038/s41467-018-06238-6 (PMC6138651; doi:10.1038/s41467-018-06238-6)
Supplement: Supplementary file 3 — Description of Additional Supplementary Files [file 41467_2018_6238_MOESM3_ESM.pdf]

Description of Additional Supplementary Files

File Name: Supplementary Software 1
Description: The kmz file for viewing the study area by Google Earth.

File Name: Supplementary Data 1
Description: The detailed information for the cobbles measured for dip directions at three locations.
